# Predicting surgical intervention in patients presenting with carpal tunnel syndrome in primary care

**DOI:** 10.2147/CLEP.S154409

**Published:** 2018-06-29

**Authors:** Claire L Burton, Linda S Chesterton, Ying Chen, Danielle A van der Windt

**Affiliations:** Arthritis Research UK Primary Care Centre, Research Institute for Primary Care & Health Sciences, Keele University, Keele, Staffordshire, UK, c.burton@keele.ac.uk

**Keywords:** carpal tunnel syndrome, prognosis, epidemiology, primary care

## Abstract

**Purpose:**

Carpal tunnel syndrome (CTS) is a symptomatic compression neuropathy of the median nerve. This study investigated the value of candidate prognostic factors (PFs) in predicting carpal tunnel release surgery.

**Patients and methods:**

This is a retrospective cohort study set in the Clinical Practice Research Datalink. Patients ≥18 years presenting with an incident episode of CTS were identified between 1989 and 2013. Candidate PF’s defined in coded electronic patient records were identified following literature review and consultation with clinicians. Time to first carpal tunnel release surgery was the primary end point. A manual backward stepwise selection procedure was used to obtain an optimal prediction model, which included all the significant PFs.

**Results:**

In total, 91,412 patients were included in the cohort. The following PFs were included in an optimal model (C-statistic: 0.588 [95% CI 0.584–0.592]) for predicting surgical intervention: geographical region; deprivation status; age hazard ratio (HR 1.02 per year, 95% CI 1.01–1.02); obesity (HR 1.23, 95% CI 1.19–1.27); alcohol drinker (HR 1.05, 95% CI 1.00–1.10); smoker (HR 1.06, 95% 1.03–1.10); inflammatory condition (HR 1.13, 95% CI 0.98–1.29); neck condition (HR 1.13, 95% CI 1.03–1.23); and multisite pain (HR 1.10, 95% CI 1.05–1.15). Although not included in the multivariable model, pregnancy (if gender female) within 1 year of the index consultation, reduced the risk of surgery (HR 0.24, 95% CI 0.21–0.28).

**Conclusion:**

This study shows that patients who are older and who have comorbidities including other pain conditions are more likely to have surgery, whereas patients presenting with CTS during or within a year of pregnancy are less likely to have surgery. This information can help to inform clinicians and patients about the likely outcome of treatment and to be aware of which patients may be less responsive to primary care interventions.

## Introduction

Carpal tunnel syndrome (CTS) is a chronic focal compressive neuropathy caused by the entrapment of the median nerve at the level of the carpal tunnel in the wrist.^1^ CTS is the most common entrapment neuropathy^2^ and is characterized by symptoms of paresthesia, dysesthesia, sensory loss, and in severe cases, weakness, and atrophy of the thenar muscle. Symptoms are usually localized to the hand but can spread proximally to the forearm, upper arm, and even shoulder.^3^ Despite usually causing relatively localized symptoms, CTS can have substantial physical, psychological, and economic consequences.^4^,^5^ Previous studies have sought to estimate the prevalence and/or incidence of CTS. Such epidemiological studies have been diverse in their approach to the populations studied and case definitions applied.^6^ The reported estimates for annual prevalence range from 3,720 to 5,700 per 100,000 per year^7^–^9^ and the reported incidence from 72 to 8,200 per 100,000 per year.^6^,^10^–^16^ CTS is generally accepted to be more common in females; the female to male ratio ranges between 0.78 and 9.66.^6^,^7^ A number of previous studies have observed the trends of prevalence or incidence over time and identified an increase.^11^,^12^,^17^

The diagnosis of CTS is generally determined by clinical history and examination findings,^18^ although electrodiagnos-tic tests are requested to confirm the diagnosis or differentiate among diagnoses.^19^ The treatment of CTS is usually defined as either surgical or conservative (nonsurgical). Local steroid injections and night splinting form the mainstay of conservative primary care interventions in CTS, as indicated by national care pathways.^20^,^21^ Patients with moderate or deteriorating symptoms following conservative treatment or sudden and severe symptoms should be referred for consideration of surgery.^22^ Carpal tunnel release surgery (CTR) is routinely carried out under local anesthetic as day surgery. Open and endoscopic approaches are used to release the flexor retinaculum. Adjuncts to the release include a tenosynovectomy, neurolysis of the median nerve, or lengthening or reconstruction of the flexor retinaculum.^23^ A review of the surgical treatment of CTS reported that 70%–90% of patients undergoing a CTR have a good outcome (definitions varied).^24^ In a retrospective cohort study over a mean follow-up of 13 years post surgery, 88% of patients were either completely satisfied or very satisfied with surgical outcome; 74% reported their symptoms had completely resolved and 1.8% (113 patients) had undergone repeat surgery.^25^ There is little evidence, however, that CTR is an appropriate initial management option for patients with mild to moderate symptoms, especially in the absence of high-quality trial evidence that conservative management is unlikely to be effective.

Episodes of CTR appear to be on the increase, with audit data from one major tertiary UK Hand Centre suggesting that referral for CTR increased over a 10-year period from 59.7 to 112 per 100,000 population per year between 1989–1999 and 2000–2011.^26^ Using Hospital Episode Statistics (HES) between 1998 and 2011, Bebbington and Furniss also observed an increase in the absolute number of patients with CTS and episodes of CTR; however, they noted a decrease in the use of surgery per diagnosis, post 2008.^27^ The increasing prevalence of the etiological factors are cited as contributing to the observed and predicted increase in the prevalence of the condition.^27^ In the UK, CTR has been labeled by some Clinical Commissioning Groups (member organizations who determine how National Health Service (NHS) funds are spent in a particular locality) as a “procedure of limited clinical value” leading to variable patient access to the procedure.^28^ The Royal College of Surgeons expressed their concern on the potential impact on patient health and well-being.^29^

The general course of CTS, prior to surgical management, is variable.^30^ We conducted a systematic review of studies summarizing evidence regarding the course and prognosis of CTS; four studies^31^–^34^ investigated the rate of surgery in patients initially treated with conservative management, reporting a range of 57%–66% of patients receiving surgery during follow-up of between 1 and 3 years. Studies published since the review have reported lower rates of surgery following local corticosteroid injection (15%–41% with follow-up between 1 and 8 years).^35^–^37^ Evidence is therefore equivocal. A narrative synthesis of the 16 studies included in the review failed to identify consistent evidence of factors predicting poor outcome (including CTR), following conservative treatment.^30^ Limitations of previous research identified by the review included a lack of studies conducted in a primary care setting, where most conservative management takes place. Design of the studies also showed wide variability with respect to characteristics of the included population, definition of CTS, assessment of prognostic factors, types of interventions provided, and types of outcome measures applied.

This study therefore aims to investigate the predictive value of candidate prognostic factors, identified through both review of the literature and discussion with clinicians, available in primary care consultation data, to predict (the first occurrence of) CTR surgery as an indicator of poor outcome of conservative management deliverable in primary care. Such evidence would be of benefit to patients, clinicians, and policy makers to inform planning of health care resources and decision-making regarding the management of CTS.

## Methods

### Setting

This was a retrospective cohort set in the Clinical Practice Research Datalink (CPRD). CPRD is a live primary care database of anonymized medical records, holding information of over 11.3 million patients from 674 practices in the UK since 1987. 4.4 million active (alive and currently registered) patients are currently contributing information to the datalink, which equates to 6.9% of the UK population.^38^ CPRD is broadly representative of the UK general population in terms of age, gender, and ethnicity.^38^ During clinical interactions, patients’ signs and symptoms, treatments and therapies, investigations, occupations, diagnoses, and appliances are assigned Read Codes, which are stored in the electronic primary care records in a retrievable and analyzable format,^39^ although in practice, these can also be recorded in free text and as such are not always retrievable. The CPRD has National Research Ethics Committee approval for observational research using primary care data, and as such no further permissions were required. The Independent Scientific Advisory Committee study protocol 14_167 was approved in September 2014.

### Study population (start point)

Individuals >18 years of age with evidence of an incident diagnosis of CTS and at least 2 years of acceptable data preceding the diagnosis were identified between 1989 and 2013, using the diagnostic Read code for CTS (F340). Patients were required to have up to standard (practice level) research quality (patient level) data in CPRD, for 2 years prior to an incident episode. The “up to standard” metric is based on continuity of recording and recording of deaths and set at the latest date at which practices met the quality criteria. The acceptable patient metric is based on registration status, the patient record itself, and a valid entry of age and gender.^38^

In order to attempt avoiding crossover between diagnosis and treatment (potentially bilaterally or a recurrent episode), only the first incident CTS episode was included, and thus, no patient with CTS was knowingly included more than once. It remained possible that a patient could be included more than once if they moved to another practice contributing to CPRD during the follow-up period, and consulted with a second episode of incident CTS after 2 years of registration, but this was considered to be unlikely. Only patients with an incident episode identified with a surgical (treatment) code were excluded, as it was not possible to identify a start point and baseline presentation for such patients.

### End point

The maximum length of follow-up was set to 3 years. Surgery occurring >3 years after the baseline diagnosis was felt unlikely to be related to the observed episode. Three years was chosen to include the presumed maximum period of 2 years, during which an episode was considered to be ongoing, plus 1 year for further referrals, investigation, and surgery. CPRD is not able to discriminate between handedness and since around 50% of patients with CTS experience bilateral symptoms, it was felt using more than a 3-year follow-up would increase the risk of including events in the contralateral hand.

### Candidate prognostic factors

Seventeen candidate prognostic factors were identified based on an appraisal of the available literature and clinical consensus work with a group of general practitioners and physiotherapists with a specialist interest in musculoskeletal health. Only those variables identifiable by Read code were included. Read code lists were either taken from an Institutional store or developed by the author (Read code lists available on request). The prognostic factors were subdivided into patient demographics (age, gender, geographical region, and deprivation score); lifestyle factors (evidence of obesity, smoking history, alcohol history, and pregnancy within 1 year of the index diagnosis of CTS); and comorbidities (affective disorders, hypothyroidism, diabetes, inflammatory conditions, neck conditions, multisite pain [including osteoarthritis], tendonitis/epicondylitis, and previous wrist trauma). Year of diagnosis was included in the unadjusted univariable analysis but not included in the multivariable model as this would have, by definition, prevented the model being used contemporaneously. Pregnancy was also not included in the multivariable model as it would limit its applicability to the presenting population.

For the multivariable prediction model, age was analyzed as a continuous variable, but the association with time to surgery was also presented in 10-year age categories for descriptive purposes. Body mass index (BMI) was dichotomized into obese and nonobese categories as it was considered clinically important to distinguish between these two subgroups. Smoking and drinking were identified in CPRD as cigarettes per day and alcohol units per week but entries were more frequently observed in the additional CPRD dataset which provided a “yes / no / unknown” outcome to “smoker” and “alcohol drinker”. These binary variables were selected as there were fewer missing data and they were considered to be clinically meaningful.

### Statistical methods

Cox proportional hazards modeling was used to determine the association between candidate prognostic factors and the first episode of surgical intervention. This time to event analysis included the censoring of patients when they received CTR, were recorded as deceased, left the practice, or the practice no longer contributed to CPRD. Proportional hazards assumptions were checked using Schoenfeld residual testing.

Univariable (unadjusted) analysis was performed to describe the crude association of each prognostic factor with outcome. All candidate factors were then entered in a multivariable model, and a backward selection procedure was used to determine the prognostic factors for the final prediction model. Prognostic factors with a *P*-value >0.1 were omitted at each step, using a conservative significance level of 0.1 to reduce the risk of missing prognostic factors with potential clinical importance.^40^ Prognostic factors eliminated were reentered in the final multivariable model with adjustment for the remaining prognostic factors to assess if they were of predictive value in the presence of other combinations of factors. The predictive performance (discrimination) of the final multivariable model was assessed using concordance statistics (C-statistics).^41^

Not all practices contribute deprivation data to CPRD, and in some cases, data were missing for lifestyle variables including BMI, alcohol use, and smoking history. Missing data were accounted for by adding a “missing” category to predictor variables. Missing data were not imputed as such data were likely to be “missing not at random” (MNAR; i.e., a patient with obesity was more likely to have a BMI recorded that someone who was not obese). Imputing data that are MNAR increases the risk of bias,^42^ and we therefore used a sensitivity analysis using complete cases only to examine the potential effects of missing data.

The number of events (cases with CTR) within the CPRD cohort was sufficiently large to allow reliable estimation of all 46 predictor parameters in the full model when considering an event per predictor parameter ratio of at least 10^43^ or even 20.^44^,^45^

## Results

In total, 91,412 patients were included in the cohort. Out of this, 18,500 (20.2%) had surgery in the 3-year period following the index presentation (absolute CPRD population rate: 1.52 episodes of surgery per 100 person-years). The median time to surgery was 221 days interquartile range (IQR 111–409). Table 1 describes the demographic and clinical characteristics of all eligible patients at baseline with an incident CTS diagnosis code and of those without any data missing from collected prognostic factors (n = 44,522). A total of 2,967 patients had a preceding incident episode coded only with a surgical code for CTR and were not included in the analysis. Similarly, 253 patients with a diagnostic code attributed on the same day as a CTR code, and 8 patients diagnosed on their “end date” had no follow-up period to observe and were not included in the analyses.

Table 2 describes the unadjusted univariable associations of each candidate prognostic factor with time to surgery. The prognostic factor with the largest effect size was pregnancy, suggesting that patients presenting within a year of their coded antenatal period, were less likely to require surgery than other females (this analysis was completed in female patients only). Diabetes, inflammatory conditions, and multisite pain appeared to be predictors of surgery on univariable analysis.

Following the manual stepwise process of selecting candidate prognostic factors, the final multivariable model was derived, as shown in Table 3. Variables included in the final model are geographical region; deprivation status; age hazard ratio (HR 1.02 per year, 95% CI 1.01–1.02); obesity (HR, 1.23, 95% CI 1.19–1.27); alcohol drinker (HR 1.05, 95% CI 1.00–1.10); smoker (HR 1.06, 95% 1.03–1.10); inflammatory condition (HR 1.13, 95% CI 0.98–1.29); neck condition (HR 1.13, 95% CI 1.03–1.23); and multisite pain (HR 1.10, 95% CI 1.05–1.15). The Harrell’s C concordance statistic for this model is 0.588 (95% CI 0.584–0.592). All variables except age, region, and deprivation met the Cox proportional hazards assumption. For these variables, the model considers the average effect over the 3-year follow-up period.

The results of the sensitivity analysis, using data only from patients who had complete predictor data (i.e., entries for deprivation, BMI, smoking, and alcohol status), are shown in Table 4. Deprivation status, alcohol status, smoking status, and neck conditions were lost from this model, suggesting these factors, particularly the lifestyle factors, may not be predictors of surgery. The Harrell’s C concordance statistic for this model was 0.587 (95% CI 0.581–0.593).

## Discussion

### Course of CTS in the observed cohort

This is a contemporary study in a large database detailing the current outcome of patients being diagnosed with CTS in a primary care setting. Twenty percent of the cohort required surgery in the 3-year period following their incident consultation, over the course of the study period from 1989 to 2013. Our recent systematic review^30^ showed widely varying estimates for the outcome of conservatively treated CTS between included cohort studies. Such a variability was likely to be due to differences between the populations observed and the definition of outcome applied (i.e., patient-recorded outcome or surgical intervention). The range (57%–66%) of patients observed to receive surgery following conservative management over a period of between 1 and 3 years^31^–^34^ was substantially higher than the figure reported in this cohort study. All four of the studies included in the review were set in secondary or tertiary hand clinics. Such populations have, in effect, already been selected due to the severity of their symptoms and/or lack of response to conservative management. This CPRD-derived cohort is likely to be more representative of the UK general practice population than cohorts observed in specialized care settings. Studies published since this review, focusing more specifically on local corticosteroid injection, have reported slightly lower rates of surgery,^35^–^37^ possibly suggesting the use of surgery post conservative management is in decline. If this is the case, understanding the prognosis of conservative management would become all the more important.

It is possible that the occurrence of surgery this study observed was underestimated by the methods applied. We included patients with a diagnostic code for CTS and had to exclude cases where the episode was recorded only using a code for surgery, as it was not possible to observe such patients from their start point (baseline) to surgery or alternative end point. Consequently, findings from this study will represent a conservative and likely underestimated proportion of the CPRD population who required surgery. Furthermore, the observed period following the index incident episode was limited to 3 years as it was felt likely that a surgical outcome after this period was unlikely to be associated with the index presentation, and possibly related to a contralateral or recurrent episode. Finally, an episode of surgery was taken to indicate that the patient’s symptoms or functional deficit had not responded to conservative treatment, or had been severe enough to warrant direct surgical consideration. This does not mean, however, that the patient without a surgical episode was necessarily symptom-free and functionally well at the end of the follow-up; consultation data are unable to provide such a level of detail.

### Predicting the risk of having a recorded episode of surgery

Despite a number of candidate prognostic factors showing a significant association with surgery, the resulting multivariable model predicts a surgical outcome only moderately better than chance, with a C-statistic of 0.588 (where perfect prediction is 1).

Univariable analysis suggests that increasing age, year of diagnosis (removed from final model as would require contemporaneous data), geographical region, obesity, a record of alcohol consumption, diabetes, inflammatory conditions, neck conditions, and multisite pain, all increase the risk of having surgery. However, on multivariable analysis, diabetes, for example, does not retain significance and the final model itself does not perform well. Although some previous studies have suggested the prognostic value of these candidate predictors, evidence regarding these prognostic factors from the systematic review was not consistent,^30^ which is in keeping with the results of this study.

On univariable analysis, the prognostic factor with the largest effect size was pregnancy (HR 0.24, 95% CI 0.21–0.28, measured in the female population). While not included in the final model (doing so would limit the application of the model to a female population), this suggests that patients presenting with CTS in pregnancy are less likely to require surgery than other nonpregnant patients. This is in keeping with a systematic review by Padua et al, which concludes that given the high rates of resolution of CTS following delivery, surgery should be reserved for cases in which conservative management fails or where functional impairment is severe or debilitating.^46^

Within the final model, factors with the largest effect size included certain geographical regions (in particular Scotland and the East Midlands) and obesity. Region is likely to be a predictor of surgery due to the variability in local care pathways and access to surgery. The inclusion of region in the model acts to control for this, as much as possible. As shown by a recent meta-analysis of 58 studies, obesity is not only a risk factor for the onset of CTS, but also a predictor of CTR (adjusted OR = 2.02, 95% CI 1.92–2.13). Obesity, as both a pathogenic cause of CTS and a predictor of severity, is likely to be due to the shape of the wrist exerting excess pressure on the median nerve.^47^ The findings from our study further suggest that obesity is associated with surgical intervention in people presenting with CTS in primary care.

### Limitations

The nature of using electronic consultation data meant that not all patients had complete datasets, for example, not all patients had a recorded BMI or smoking status. As deprivation linkage was available in practices in England only and not the whole of the UK, this again limited the number of patients with complete data substantially (by 46,890 patients, or 51% of the original cohort). The sensitivity analysis performed using only patients with complete data suggests deprivation, alcohol use, and smoking history may not be significant predictors of surgery; however, these results may be biased due to selective missing data (MNAR). Patients with evidence of such Read codes, in particular related to lifestyle variables, are likely to be a selected sample rather than representative of the total presenting population; for example, a patient who is obese is more likely to have a documented BMI than a patient who is not obese.

The quality of research is dependent on the completeness and accuracy of the data it utilizes. While validation studies of the CPRD have shown a high positive predictive value for some diagnoses,^38^ the sensitivity and specificity of diagnoses are largely unreported.^48^ It remains possible that the coding of a diagnosis of CTS, and indeed some prognostic factors, was inaccurate or absent. For example, a patient with a history of neck pain may not have received a Read code and hence not have been identified by the study. Patients with chronic conditions, such as diabetes, due to the regular structured follow-up they receive in primary care, may have been more likely to receive a clinical code than someone with a pain-related problem. Such underreporting may contribute to the limited performance of the final predictive model.

The study was also reliant on any surgical episode being captured by clinical coding in the database. As procedures now routinely take place outside of the secondary care environment (from circa. 2008 when the “any willing provider” concept was introduced), it was felt that HES would underestimate the episodes recorded, and hence primary care documentation was used. This relies again on the accurate and precise coding of correspondence into primary care.

Other factors may explain the poor predictive performance of the model. The most probable reason is that potentially important prognostic factors for future surgery could not be measured in CPRD, which include symptom duration and clinical tests such as a positive Phalen’s test and thenar atrophy.^30^ While Read codes do exist for Phalen’s test and thenar atrophy, pilot work demonstrated that they were seldom used and therefore unlikely to provide reliable data when extracted from CPRD. Although geographical region was incorporated into the model as a proxy for the local variability in access to CTR, this will not fully reflect decisions at the level of commissioning health care. Also, the data cannot take into account patient preference and practitioners’ referral and management behavior, which may be further possible reasons for the low predictive performance (C-statistic) of the model.

### Implications for clinical practice

The aim of this study was to use consultation data to predict the risk of an episode of surgery in patients presenting in primary care with CTS, by developing a prognostic model. In order for a prognostic model to have good clinical utility, it should be generalisable to populations that have similar ranges of predictor variables, use unambiguous definitions of predictors and outcomes reproducible in clinical prac-tice, and be tested in impact studies in order to estimate the effect of using the model on physicians’ behavior (treatment decision-making) and the clinical and cost-effectiveness compared to care without the use of the model.^49^ While CPRD is generalizable to the UK primary care population^38^ and utilizes Read codes, which represent the language used in clinical practice, this model needs to be further developed to improve its predictive performance, using data from high-quality prospective cohort studies with sufficient information regarding potential prognostic factors, and subsequently tested for generalizability and clinical impact. The model, however, does confirm the potential predictive value of several prognostic factors, including obesity, lifestyle factors, other musculoskeletal pain, and comorbidity in a large representative primary care population.

## Conclusion

Predicting the course and outcome of CTS presenting in primary care remains challenging. It was not possible to derive a prognostic model with strong predictive performance using data from routinely collected electronic health care data. Future research needs to include more detailed information regarding the clinical history and physical examination findings in patients with CTS to improve predictive performance.

## Figures and Tables

**Table 1:** Description of cohort and cohort without missing data

	All patients		Patients without missing data
Participants, n	91,412		44,522
Patients with a coded episode of surgery n (%)	18,500 (20.24)		8,971 (20.15)
Practice	685 practices contributed		383 practices contributed
Year of baseline diagnosis, median (IQR)	2006 (2002–2010)		2007 (2003–2010)
Year of surgery, median (IQR)	2007 (2004–2011)		2008 (2004–2011)
Time to surgery in days, median (IQR)	221 (111–409) max 3 years		249 (118–559) max 3 years
Follow-up time in days, median (IQR)	1,095.75 (385–1,095.75)		1,095.75 (370–1,095.75)
**Demographics**			
Age at diagnosis, median (IQR)	53.00 (42.00–66.00)		53.96 (43.20–66.31)
Age group, n (%)			
18–29	4,713 (5.16)		1,810 (4.07)
30–39	13,741 (15.03)		6,504 (14.61)
40–49	19,366 (21.18)		9,504 (21.35)
50–59	21,597 (23.63)		10,825 (24.31)
60–69	13,964 (15.28)		69,333 (15.57)
>70	18,032 (19.73)		8,946 (20.09)
Female, n (%)	63,194 (69.13)		32,030 (71.94)
Geographical region, n (%)			
North East	1,460 (1.6)		929 (2.09)
North West	9,637 (19.59)		5,928 (13.31)
Yorkshire and The Humber	3,984 (10.54)		2,248 (5.05)
East Midlands	3,583 (3.92)		1,613 (3.62)
West Midlands	8,281 (9.06)		5,193 (11.66)
East of England	10,191 (11.15)		6,230 (13.99)
South West	8,841 (11.15)		5,931 (13.32)
South Central	10,832 (11.85)		5,876 (13.20)
London	8,270 (9.05)		5,232 (11.75)
South East Coast	9,483 (10.37)		5,342 (12.00)
Northern Ireland	2,371 (2.59)		–
Scotland	6,366 (6.96)		–
Wales	8,113 (8.88)		–
Deprivation score			
1 (least)	13,878 (15.18)	23.82^d^	10,631 (23.88)
2	14,041 (15.36)	24.10^d^	10,507 (23.60)
3	11,800 (12.91)	20.25^d^	8,981 (20.17)
4	10,594 (11.59)	18.18^d^	8,241 (18.51)
5 (most)	7,950 (8.70)	13.65^d^	61,362 (13.84)
Unknown	33,149 (36.26)	–	–
**Lifestyle factors**			
Body mass index^a^ (Kg/m^2^), median (IQR)	27.2 (23.9–31.2)		27.1 (24–31.2)
<30	54,209 (59.30)	67.24^d^	30,480 (68.46)
≥30	26,410 (28.89)	32.76^d^	14,042 (31.54)
Unknown	10,793 (11.81)	–	–
Ever drinker, n (%)			
No	10,968 (12.00)	13.94^d^	6,034 (13.55)
Yes	67,736 (74.10)	86.06^d^	38,488 (86.45)
Unknown	12,708 (13.90)	–	–
Ever smoker, n (%)			
No	50,924 (55.71)	62.95^d^	28,432 (63.86)
Yes	29,967 (32.78)	37.05^d^	16,090 (36.14)
Unknown	10,521 (11.51)	–	–
Pregnancy (in female patients)^b^, n (%)	3,869 (4.23)		1,908 (5.96)
**Comorbidities**			
Affective disorder^c^, n (%)	4,576 (5.01)		2,244 (5.04)
Hypothyroidism^c^, n (%)	1,904 (2.08)		951 (2.14)
Diabetes^c^, n (%)	1,795 (1.96)		1,012 (2.27)
Inflammatory condition^c^, n (%)	851 (0.93)		426 (0.96)
Neck condition^c^, n (%)	2,371 (2.59)		1,113 (2.50)
Multisite pain (including osteoarthritis)^c^, n (%)	7,799 (8.53)		3,854 (8.66)
Tendonitis/epicondylitis^c^, n (%)	850 (0.93)		421 (0.95)
Wrist trauma^c^, n (%)	615 (0.67)		294 (0.66)

**Notes:**

a Closest recorded value preceding the baseline diagnosis.

b Identified between 1 year prior to baseline and baseline.

c Identified between 2 years prior to baseline and baseline.

d Percentage of patients, excluding the “unknown” category.

e Censored at the episode of surgery.

**Abbreviations:** IQR, interquartile range.

**Table 2:** Unadjusted univariable associations of each candidate prognostic factor with time to surgery (all patients)

Candidate prognostic factors	Hazard ratio	95% CI
Age at diagnosis^a^	1.01	1.01–1.02
Age group^a^		
18–29	1	
30–39	1.72	1.59–1.92
40–49	2.28	2.05–2.52
50–59	2.67	2.41–2.95
60–69	2.70	2.43–3.00
>70	3.33	3.00–3.68
Gender (female)^a^	0.97	0.94–1.00
Year of diagnosis^a^	1.02	1.01–1.02
Geographical region^a^		
London	1	
North East	1.20	1.05–1.36
North West	1.30	1.21–1.39
Yorkshire and The Humber	1.23	1.13–1.35
East Midlands	1.62	1.48–1.76
West Midlands	1.43	1.34–1.54
East of England	1.11	1.04–1.19
South West	1.21	1.12–1.29
South Central	1.31	1.23–1.41
South East Coast	1.23	1.15–1.32
Northern Ireland	1.11	1.00–1.24
Scotland	1.69	1.57–1.82
Wales	1.28	1.19–1.37
Deprivation score^a^		
1 (least)	1	
2	0.96	0.912–1.01
3	0.99	0.94–1.05
4	0.92	0.87–0.98
5 (most)	0.96	0.90–1.02
Not known	1.00	0.96–1.04
Obesity		
Not obese (BMI <30)	1	
Obese (BMI ≥30)	1.21	1.17–1.25
Not known	0.87	0.83–0.91
Ever alcohol drinker		
No	1	
Yes	1.06	1.01–1.11
Not known	0.94	0.88–0.99
Ever smoker		
No	1	
Yes	0.97	0.94–1.01
Not known	0.99	0.94–1.03
Pregnancy (if gender = female)^a^	0.24	0.21–0.28
Affective disorder	0.96	0.90–1.03
Hypothyroidism	1.06	0.96–1.17
Diabetes	1.26	1.14–1.39
Inflammatory condition	1.26	1.10–1.45
Neck condition	1.15	1.06–1.25
Multisite pain (including osteoarthritis)	1.22	1.16–1.27
Tendonitis/epicondylitis	1.02	0.88–1.18
Wrist trauma	1.04	0.87–1.24

**Notes:** *P*-value obtained from each group compared to the referent group.

a Variable does not fit the proportional hazard assumption and therefore presents the average effect over the follow-up period.

**Abbreviation:** BMI, body mass index.

**Table 3:** Final multivariable prediction model of prognostic factors associated with time to surgery (all patients)

Prognostic factors	Hazard ratio	95% CI
Age at diagnosis^a^	1.02	1.01–1.02
Geographical region^a^		
London	1	
North East	1.20	1.05–1.36
North West	1.30	1.22–1.40
Yorkshire and The Humber	1.26	1.15–1.38
East Midlands	1.65	1.52–1.80
West Midlands	1.43	1.33–1.53
East of England	1.09	1.02–1.18
South West	1.16	1.08–1.25
South Central	1.31	1.22–1.40
South East Coast	1.20	1.12–1.29
Northern Ireland	1.18	1.06–1.33
Scotland	1.78	1.65–1.93
Wales	1.32	1.22–1.43
Deprivation^a^		
1 (least deprived)	1	
2	0.96	0.91–1.01
3	1.00	0.95–1.06
4	0.94	0.89–1.00
5 (most deprived)	0.98	0.92–1.04
Unknown	0.92	0.87–0.96
Obesity		
Not obese	1	
Obese	1.23	1.19–1.27
Unknown	0.89	0.84–0.94
Ever alcohol drinker		
Never drinker	1	
Ever drinker	1.05	1.00–1.10
Unknown	1.08	1.01–1.15
Ever smoker		
Never smoked	1	
Ever smoked	1.06	1.03–1.10
Unknown	1.01	0.97–1.07
Inflammatory condition	1.13	0.98–1.29
Neck condition	1.13	1.03–1.23
Multisite pain	1.10	1.05–1.15

**Notes:** *P*-value obtained from each group compared to the referent group.

a Variable does not fit the proportional hazard assumption and therefore presents the average effect over the follow-up period.

**Table 4:** Sensitivity analyses: final multivariable prediction model of prognostic factors associated with time to surgery, only including patients without missing data

Prognostic factors	Hazard ratio	95% CI (lower)
Age at diagnosis^a^	1.02	1.01–1.02
Geographical region^a^		
London	1	
North East	1.39	1.27–1.52
North West	1.19	1.06–1.34
Yorkshire and The Humber	1.80	1.60–2.03
East Midlands	1.45	1.32–1.58
West Midlands	1.21	1.11–1.32
East of England	1.25	1.14–1.36
South West	1.35	1.24–1.48
South Central	1.30	1.19–1.42
South East Coast	1.28	1.09–1.50
Obesity		
Not obese	1	
Obese	1.21	1.16–1.26
Inflammatory condition	1.37	1.14–1.64
Multisite pain	1.08	1.00–1.15

**Note:**

a Variable does not fit the proportional hazard assumption and therefore presents the average effect over the follow-up period.
